# Tswana traditional health practitioners’ perspectives on the management of diabetes and hypertension: a qualitative study using focus group discussions

**DOI:** 10.11604/pamj.2019.34.93.19112

**Published:** 2019-10-16

**Authors:** Ebenezer Frimpong, Manimbulu Nlooto

**Affiliations:** 1 The Discipline of Pharmaceutical Sciences, School of Health Sciences, University of KwaZulu-Natal, Durban, South Africa

**Keywords:** Diabetes, hypertension, herbal mixtures, traditional health practitioners

## Abstract

**Introduction:**

The literature suggests the involvement of Traditional Health Practitioners (THPs) perspectives in treating diabetes and hypertension in Africa. This study sought the perspectives of Tswana THPs in the management of both diabetes and hypertension.

**Methods:**

Using a semi-structured interview guide, four Focus Group Discussions (FGDs) sessions were held with 40 THPs; FGD1 (12) FGD2 (6); FGD3 (13) and FGD4 (9) who were purposely selected from Bojanala and Dr. Ruth Sekgopomati Districts in the North-West Province of South Africa.

**Results:**

Tswana THPs perceived diabetes as a “sugar” disease and described hypertension as a disease associated with the abnormal flow of blood in a patient's body. In addition, some of the signs and symptoms of both diabetes and hypertension mentioned by Tswana THPs agreed with scientific literature. Tswana THPs employed the use of the following plants: borago officinalis, ziziphus mucronata, hypoxis hemerocallidea, sutherlandia frutescens, senna italica, urginea sanguinea and eucalyptus globulus in the management of diabetes and hypertension.

**Conclusion:**

Some of the medicinal plants employed by THPs in the management of both diabetes and hypertension has been proven scientifically to be effective against these chronic conditions.

## Introduction

In most African communities where access to hospitals and clinics remains a challenge, patronage of Traditional Medicine (TM) with the help of Traditional Health Practitioners (THPs) offer people the opportunity to manage certain diseases affecting their wellbeing and health in general [1]. According to the World Health Organization (WHO), "traditional medicine refers to health practices, approaches, knowledge and beliefs incorporating plant, animal and mineral-based medicines, spiritual therapies, manual techniques, and exercises applied singularly or in combination to treat, diagnose and prevent illnesses or maintain well-being" [2]. Furthermore, WHO defines Traditional Health Practitioner(THP) as, “a person who is recognised by the community where he or she lives as someone competent to provide health care by using plant, animal, mineral substances and other methods based on social, cultural and religious practices” [3]. In South Africa, a study reported that there were four groups of THPs recognized by the South African traditional healers act, namely: herbalists (izinyanga or amaxhwele), diviners (izangoma, umthandazi or amagqirha), traditional surgeons (iingcibi) [4]. The role played by THPs in the treatment and management of these two diseases (diabetes and hypertension) across the African continent has been documented [5, 6]. A research carried out in South Africa revealed the treatment of diabetes among THPs and faith healers in the northern province of South Africa [5]. Similarly, a study conducted in Zambia reported the treatment of hypertension among THPs in Zambia [6]. The two leading causes of cardiovascular diseases and their complications are diabetes and hypertension. Globally, it has been estimated that 1.39 billion people are affected by hypertension [7] while 300 million people are estimated to be diabetic by the year 2025 [8]. The present study sought Tswana THPs perspectives about the management of diabetes and hypertension. The specific objectives were: to determine Tswana THPs cultural understanding of both diabetes and hypertension; to determine the Tswana THPs description of clinical features for both diabetes and hypertension; to determine ethnopharmacological and treatment modalities employed by Tswana THPs in the management of both diabetes and hypertension.

## Methods

**Study design and setting:** an exploratory descriptive study, using four FGDs, was conducted among THPs in urban, traditional and farmland areas in the Bojanala and Dr. Ruth Sekgopomati Districts of North West district in South Africa to obtain quantitative and qualitative data. It is significant to point out that FGDs are used to gather opinions in qualitative research [9]. In the Bojanala District, FGD session 1 was held in the living room of a traditional leader in Boitekong, while FGD session 2 was held in the local community hall in Montsana. FGD sessions 3 and 4 in Dr. Ruth Sekgopomati Districts (Pampierstadt and Manthe) were held in the living rooms of THPs leaders.

**Study population and sample:** purposive sampling was used to identify THPs who could provide information based on their availability and willingness to co-operate [10]. The four FGD sessions (12 in FGD1); (6 in FGD2); (13 in FGD3) and (9 in FGD4) had no more than 14 subjects based on the recommendation of Gill P *et al.* [11]. The inclusion criteria for participants was a minimum of aged 18 years with more than one year experience in managing diabetes and hypertension. Those eligible THPs with less than one-year experience in the management of both diabetes and hypertension were excluded. With regards to recruitment and selection of THPs leaders of various associations of THPs in the area of study (North West Province, South Africa) were contacted to obtain their support and assistance in making contact with appropriate practitioners. Furthermore, they helped to recruit eligible THPs to join FGDs held during business hours in the respective districts.

**Ethics approval:** this study obtained approval from the Biomedical Research Ethics Committee (BREC) of the University of KwaZulu-Natal under reference number BE 567/17. Participation in this study was voluntary for THPs included in the final analysis. The purpose of this study was explained to the participants and they were allowed to ask questions before each FGD session started. Those THPs who volunteered to be part of the study gave written consent before joining FGDs.

**Data analysis:** there were simultaneous data collection and analysis with the help of two research assistants who were fluent in the Tswana language, the FGDs were audiotaped and transcribed verbatim. The raw transcribed data were analysed following Tesch's recommendation for identifying themes and sub-themes as presented in a table below with the help of independent qualitative research experts. A meeting was held between the principal researchers to discuss the codes identified after data analysis [12]. The quantitative data were analysed using SPSS version 2.0 and thematic content analysis was used to analysed qualitative data [13, 14]. Descriptive statistics were used to present socio-demographic characteristics of participants expressed in frequency and percentage; mean values with standard deviation were used for age and years of work experience.

## Results

Table 1 presents the socio-demographic characteristics of participants included in this study. Majority of the respondents recruited for the study were females (27/40, 67.5%). The mean age and years of work experience were 42.87 (13.69) and 11.29 (10.58) respectively. Majority of the participants were single (22/40, 55%); had high school education (21/40, 52.5%) and stated following both traditional and Christian religions 20/40 (50%). Many THPs 31/40 (77.5%) in this study used both divination and herbal practices. Most of the participants 36/40 (90%) were registered with both the South African Traditional Health Practitioners Council (SATHPC) and the local district associations (THA). It is significant to point out that 8 themes and 15 sub-themes emerged from the study (Table 2).

**Table 1:** Socio-demographic characteristics of THPs purposely selected for this study

Variables	Mean ± SD	n %
Age(years)	42.87(13.69)	NS
Years of experience	11.29(10.58)	NS
Gender	Female	27(67.5)
	Male	12(30)
	Not specified	1(2.5)
Marital status	Cohabitation	2(5.0)
	Married	13(32.5)
	Single	22(55)
	Widow	1(2.5)
Religion	Christian	5(12.5)
	Traditional	15(37.5)
	Traditional and Christian	20(50)
Education	Nil	1(2.5)
	Primary school	5(12.5)
	Tertiary	11(27.5)
	High school	21(52.5)
	Other (middle school)	2(5.0)
Kind of practice:	Both divination and herbal	31(77.5)
	Divination	9(22.5)
Type of Practicing	Fulltime	38(95)
	Part time	2(5.0)
Place of practice	Home	40(100.0)
	Home and market	0(0.0)
	Office	0(0.0)
Registration (SATHPC)	No	4(10.0)
	Yes	36(90.0)
Registration (THA)	No	4(10.0)
	Yes	36(90.0)

Legend a: SATHPC (South African Traditional Health Practioners Council); National body; b: THA (Traditional Healers Association; Local association in respective district; c: SD (Standard deviation); d:NS (Not stated)

**Table 2:** Major themes and sub-themes

Theme 1: Perceptions towards diabetes and hypertension	Understanding diabetes and hypertension Is diabetes and hypertension curable or manageable Identifying a person with diabetes and hypertension Perceived causes of diabetes and hypertension Perceived major complications of diabetes and hypertension
Theme 2: Treatment modalities for diabetes and hypertension	Ethnopharmacological interventions Non pharmacological interventions
Theme 3: Prescribing practices for diabetes and hypertension	Severity of a patient’s condition, high/low concentration of a mixture and age
Theme 4: Effectiveness for prescribed medications	TM vs western medications
Theme 5: Concurrent use of prescribed medicines with western medications to avoid contraindications	Encouragements and instructions to follow when on these two medications Avoidance of taken TM and western medication simultaneously
Theme 6: Feedback and monitoring	Feedback report from patients
Theme 7: Challenges faced in the management of diabetes and hypertension	Inaccessibility of medical tools for diagnosis
Theme 8: Recommendations to improve diabetic and hypertensive care in South Africa	Collaboration with biomedically health professionals The need for a two-way referral system of patients Endorsement and recognition from government

**Tswana THPs perceptions towards diabetes and hypertension:** THPs perspectives in the management of diabetes and hypertension are based on their understanding of these two diseases whether it is curable or manageable. Besides, how they were able to identify a patient affected by diabetes and hypertension was also taken into consideration. Last but not the least, Tswana THPs emphasized on the perceived causes and major complications of these two diseases.

**Understanding diabetes and hypertension:** most Tswana THPs believed that diabetes is a “sugar disease” and described hypertension as a disease associated with a patient having an abnormal flow of blood in a patient’s body. *"It is a sugar disease; the basic level of sugar is not the way is supposed to be in one's blood.”* Female 1, GP1. *“It is a sugar disease…”* Female 3, GP4. *“Hypertension is when there is a lot of pressure in your blood system, your blood is not flowing according to a normal way.”* Female 2, GP4.

**Diabetes and hypertension are they curable or manageable?** Most of the THPs were of the assertion that diabetes and hypertension could be cured following an appropriate treatment regimen. *"Yes, both diseases are curable, with relevant treatment.” Female 2, GP2.* It is significant to point out that all the participants in (GP2) voiced out that they agreed with what she said. *“Yes. They are curable.”* ***All the THPs in GP4. *“Yes, these two diseases are curable.”* ***All the THPs in GP 3. *“Both sugar diabetes and hypertension can be brought under control.”* *** All the THPs in GP1.

**Identifying a person with diabetes and hypertension:** majority of the THPs based their diagnosis on the knowledge of the signs and symptoms of these two diseases or signs and symptoms reported by patients seen by them. Tswana THPs mentioned the following: frequent urination, excessive sweating, frequent thirst, excessive desire to eat, as possible signs and symptoms of the two diseases. A few relied on BHPs and ancestral spirits for diagnosis. *“A person suffering from diabetes urinates now and then."* Female 1, GP4. *“Diabetic person sweats a lot.”* Male 2, GP3. *"There are symptoms that we pick up when a person has diabetes, firstly that person normally has dry skin, also his/her mouth dries up. A person with diabetes is always hungry and he/she gets extremely weak when he/she is hungry, and his/her hard palate is also very dry.”* Female 8, GP1. *“The person who suffers from hypertension has similar symptoms as the one with diabetes, but also sweating is the most common symptoms just like a TB patient. Just like diabetes a person suffering from hypertension has a very low sex desire. Hypertension sufferers are normally impatient and short-tempered as well.”* Female 1, GP 1. *“Once the clinic or doctor has confirmed we can start with the treatment.”* Male 2, GP1. *“Our knowledge and also guidance from our ancestors.”* Female 2, GP2.

**Perceived causes of diabetes and hypertension:** Tswana THPs stated that poor diets (consumption of excess sugar, salt, fatty foods, and red meat) are the perceived causes of both diabetes and hypertension. *“They must avoid fatty/oily food. Salty and spicy food.”* Female 3, GP1. *"They must avoid meat, especially fatty meat, they must also avoid red meat if it is chicken they must remove the skin."* Female 4, GP3.

**Perceived complications of diabetes and hypertension:** Tswana THPs indicated poor eyesight, wounds associated with diabetic patients that do not get healed, amputation, stroke and erectile dysfunction as some of the perceived medical complications for diabetes. *“With sugar diabetes is…once you can have a minor scratch or a wound…you don't get healed.”* Female 4, GP1. *“Hypertension leads to stroke.”* Male 3, GP3. *“In men it causes erectile dysfunction.”* Female 1, GP2.

**Treatment modalities:** Tswana THPs employed both ethnopharmacological and non-pharmacological treatment approaches in the management of these two diseases

**Ethnopharmacological interventions:** Tswana THPs employed the use of herbal mixtures in the management of both diabetes and hypertension. *“We use the following herbs, for both diabetes and hypertension, both diseases normally go together, meaning often you find a person suffering from diabetes also having hypertension as well. So we use **ditserempepe, matlhare a se-rooiboom, aloe** and **mositakgomo**.”* Female 2, GP2. *"For treatment, I mix **lekgala,** and **lerumo la madi,** I also take **mosibeletswane** and boil it, afterward I pour in two-litre bottle for the patient to drink at home."* Male 1, GP4. Tswana THPs recommended the use of herbal mixtures with the composition of these medicinal plants *borago officinalis, ziziphus mucronata, hypoxis hemerocallidea, sutherlandia frutescens, senna italica, urginea sanguinea and eucalyptus globulus.*

**Non-pharmacological interventions:** Tswana THPs stated the significance of exercise in the management of both diabetes and hypertension. *“They must exercise regularly, they must take care of their bodies.”* Female 1, GP3.

**Prescribing practices:** Tswana THPs prescribed dosage leaned on the severity of a patient’s health condition and a patient’s ability to consume a certain amount of herbal mixtures.

**The severity of a patient's condition, high/low concentration of a mixture and age:** THPs in this study relied on the age of a patient, severity of patients and the concentration of prescribed herbal mixtures. *"It will depend and his/her condition, he/she might drink a quarter of a cup once, twice or three times a day."* Female 4, GP1. *"Yes, we do consider different age groups of patients as well as their conditions like pregnant women."* Female 3, GP3.

**Effectiveness of prescribed TM:** Tswana THPs argued about the effectiveness of their prescribed herbal mixtures compared to western medications.

**TM vs western medications:** THPs believed that their prescribed herbal mixtures had no side effects when taken by their patients. *"No, our traditional medication does not have any side effects…."* Female 2, GP2. *"There are no side effects."* Male 2, GP4.

**Concurrent use of both prescribed medicines and western medications to avoid contraindications:** Tswana THPs were not against taking these two forms of medications but advised their patients to take these two forms of medications (TM and conventional orthodox medication) but different times during the day to avoid contraindications.

**Encouragements when on these two forms of medications (prescribed herbal mixtures and western medications):** Tswana THPs offered encouragements and advice to patients on both their prescribed medicines and western medications for the management of these two diseases. *"Yes, they can take their herbal mixtures with western medication."* Male 3, GP3.

**Avoidance of taken TM and western medication simultaneously:** "If they take their clinic in the morning advise them to take their traditional medication in the evening or during the day." Male 3, GP2. "Also there must be a space between traditional medication and western medication, for example, if the patient takes the western one in the morning, then he/she must take the traditional one in the evening, alternatively the patient must ensure that there's four hour interval between the time he/she took the one medication before he/she can take another one. "Female 3, GP3.

**Feedback and monitoring of patients:** Tswana THPs acknowledged the significance of monitoring the progress of the patients seen by them.

**Positive feedback report from patients:** some of the THPs contended that they do follow-ups by calling their patients over the phone. *"There's constant communication between us and our patients, they always tell us and also we can see them, again often when they go for their check-ups at the clinic, they are always told whether their conditions have improved or not."* Female1, GP4.

**Challenges faced in managing these two diseases:** Tswana THPs spoke about the lack of scientific tools to help them with diagnostic purposes.

**Inaccessibility of medical tools for diagnosis:** Tswana THPs bemoaned inaccessibility of scientific tools in the management of both diabetes and hypertension. *"Western doctors have the equipment to check whether a patient is diabetic or not, with us, we only depend on signs."* Male 2, GP3. *"The only thing is that they have the equipment to detect these diseases and we only rely on the signs and guidance from our ancestors."* Male 3, GP2.

**Recommendations to improve diabetic and hypertensive care in South Africa:** Tswana THPs highlighted the significance of collaboration with BHPs to assist in the management of both diabetes and hypertension. The proposed partnership with BHPs was intended to assist in managing diabetes and hypertension.

**Collaboration with biomedically health professionals:** "I believe that if all South African medical health practitioners both traditional and western, must work together, traditional healers must also be allowed to see and treat patients in hospitals, I think that can improve not only diabetic and hypertension care but the health care in general." Female 3, GP4.

**The need for a two-way referral system of patients:** Tswana THPs suggested the implementation of a two-way referral system of patients in the management of both diabetes and hypertension. They believed that BHPs were the ones against the two-way referral system. *"The clinic and western doctors seem to be the ones who have problems to refer patients to us."* Female 2, GP3.

**Endorsements and recognition from the government:** Tswana THPs appealed to the government to give them the same attention as the BHPs working in the hospitals. *"Government must also intervene because we are realizing that western doctors are highly respected as opposed to us, African healers. We always give solutions to some of the health problems in this country, but we hardly get any recognition or credit."* Female 3, GP1.

## Discussion

Tswana THPs in this study description of diabetes as a "sugar disease" agrees with a similar study conducted in the Northern Province of South Africa where THPs described diabetes as a sugar disease [5]. Regarding hypertension, Tswana THPs associated the disease with a patient having an abnormal flow of blood in his or her body. This is in agreement with a research carried out by Goma *et al.* in Zambia where some of the THPs in Lusaka described hypertension as a disease associated with blood flowing fast in a patient’s body [6]. Tswana THPs in this study had varied opinions on whether hypertension and diabetes were curable or could only be managed. The assertion about the curability of diabetes and hypertension by THPs in FGDs 2, 3 and 4 is in agreement with the statements of THPs in the Northern Province of South Africa which indicated that diabetes was curable using African Traditional Medicine [5]. In a similar study conducted in Nigeria, THPs believed that hypertension was curable [15]. However, in FGD 1, the THPs stated that diabetes and hypertension could only be managed but not cured completely. THPs in FGD 1 assertion that these two diseases cannot be cured completely is in agreement with scientific literature as opposed to the statements made by THPs in FGDs 2, 3 and 4 which seem to suggest that these two diseases could be cured completely following appropriate treatment regimen [16, 17]. It is therefore worth noting that participants were confident in their submissions regarding the management of diabetes and hypertension.

Most of the THPs in this study based their diagnosis on the signs and symptoms reported by patients seen by them, while a few relied on ancestral spirits for diagnosis. A similar study conducted in Nkonkobe Municipality in South Africa reported that THPs relied on the signs and symptoms reported by their patients for diagnosis [18]. This finding may suggest that THPs in this study relied on the signs and symptoms reported by patients to affirm their diagnosis. Signs and symptoms (frequent urination, excessive sweating, frequent thirst, excessive desire to eat) reported by THPs in this study are in agreement with those signs and symptoms commonly reported in the scientific literature about diabetes and hypertension [19, 20]. Some of the THP's in this study stated with regards to a spiritual diagnosis that, upon receiving patients to their shrine "bidime" in "Tswana language" during consultations, ancestors revealed to them the problem troubling an individual and appropriate measures to be undertaken to improve his or her condition. This finding above is in agreement with another study which stated that ancestors have the power to heal [21]. Furthermore, indigenous epistemology suggests that an individual becomes ill as a result of weaknesses in his or her protective (spiritual) immunity [22].

Perceived causes (too much sugar and salt, excessive consumption of red meat and alcohol, and lack of regular exercises) of diabetes and hypertension described by THPs in this study are in agreement with the scientific findings commonly reported for diabetes and hypertension [23, 24]. Regarding perceived complications of diabetes and hypertension, the THPs specifically mentioned the following: erectile dysfunction, poor eyesight, wounds associated with diabetic patients that do not get healed, leading to stroke and amputation, this being the last intervention for limb preservation in diabetic patients with foot ulcers [25]. A multi-ethnic cohort study in the USA revealed a diabetic retinopathy prevalence rate of 33.2% among the studied population [26]. Erectile dysfunction, as stated by THPs in this study, is one of the major complications associated with diabetic patients and agrees with scientific literature. The prevalence rates of erectile dysfunction reported among diabetic men conducted in the following countries were as follows: USA (more than 50%), Saudi Arabia (80-90%), Netherlands (41%) and Mexico (30-80%) [27].

Although most of the THPs were not ready to disclose information about the herbal formulation they used to manage the two diseases, some named the following herbs and plants: buffalo-thorn (*mokgalo*), eland's-pea (*sebetebete*), sutherlandia (*lerumo-la-madi/phetola/mhetola*), blue-gum tree (*bluekom*), red slangkop (*sekaname*), African potato (labatheka), star-flower (*tshuko-ya-poo*), aloe vera (*mokgopha*), dwarf buffalo-thorn (*sekgalofatshe*). To prepare a herbal mixture some said they took fresh aloe, a handful of dried powder form of star-flower, a handful of *tlhokwa-la-tsela* (yellow medicinal grass-like plant) and a secret plant (often given a funny term) known only to them. A mixture of the above-mentioned plants was poured into a pot and allowed to boil. The boiled herbal mixture is taken off the fire and allowed to cool off before they poured into a 2-litre clean bottle to be given to patients. Scientific findings have revealed the potency of some of the named plants reported in this study for regulating blood sugar levels and decreasing blood pressure in both humans and animals. Approximately sixty type 2 diabetic patients in India who were administered with a supplement (aloe vera gel powder, 100/200mg) daily over three months recorded a significant reduction in their blood glucose and blood pressure levels [28]. Crude leaf extracts of starflower (*borago officinalis*) administered in rabbits caused a decrease in both atrial force and rate of contractions in their hearts [29]. Experimental results revealed a decrease in intestinal glucose uptake (p<0.001 at 1-hour intervals) in diabetic wistar rats when the animals were administered with *sutherlandia frutescens* for over eight weeks [30]. In a similar study, an aqueous extract of African potato *(hypoxis hemerocallidea*) (100-800 mg/kg p.o) administered to streptozin diabetic rats caused a reduction in the animal's blood glucose concentrations (30.20% and 48.54%) [31]. In vitro studies using 0.2% 2, 2-diphenyl-2-picrylhydrazyl (DPPH) assay revealed antioxidant properties exhibited by acetone extracts of the root of elands pea (*Senna italica*) [32]. Buffalo-thorn (*ziziphus mucronata*), red slangkop (*urginea sanguinea*), blue gum tree (*eucalyptus globulus*), were also reported to be used by THPs in South Africa to manage diabetes and hypertension [33-35].

THPs in this study revealed that lifestyle modifications on the part of patients were a major contributor to manage these two diseases, with brisk walking having been established to be effective in reducing arterial blood pressures [36]. A study reported that hypertensive patients (under 8 weeks stress management programme) recorded a significant reduction in systolic blood pressures, even without taken their antihypertensive medications within that period [37]. THPs expressed confidence in their prescribed TM and were not aware of any side effects, with the dosage given depending on the severity of a patient's condition. This is in agreement with a similar study conducted in Kenya, where THPs stated that their prescribed herbal mixtures had no side effects [38]. Some of the THPs in this study were not against the concurrent use of TM and conventional orthodox medication by their patients. However, instructions were given regarding different times their prescribed medications should be taken to achieve the best possible health outcomes. Interestingly, studies reported that to avoid adverse effects regarding the use of both TM and conventional medications, these forms of medications should be taken at different times by a patient [39]. Some of the THPs in this study believed that extra care should be taken when prescribing medications for pregnant women, as strong herbal mixtures could affect the fetus. They gave an example of how some of their clients (women) who had taken birth control pills concurrently with their prescribed medications fell pregnant. Tswana THPs made it a priority to monitor the health conditions of their patients after they had given them medical assistance. In this study, some of the THPs who had the phone numbers of their clients called them to find out whether their health conditions had improved. Regarding the issue of collaboration between THPs and BHPs to assist in managing these two conditions, they were not against this initiative but bemoaned the lack of co-operation from BHPs. Similarly, a study reported the reluctance of BHPs in referring patients to THPs for cancer treatment in KwaZulu-Natal [40]. They confirmed the significance of collaboration between THPs and BHPs to improve diabetic and hypertensive care in South Africa. Most of the THPs were willing to learn to use scientific tools, such as a sphygnometer and glucometer, for diagnosing patients affected by these chronic conditions.

**Limitations of the study:** the study took place in two districts in the North West Province, and the findings, therefore, may not be generalized for the entire population of THPs in the North West province and the rest of South Africa. Medicinal plants available in the two districts under study may not be found elsewhere in other parts of South Africa.

**Implications of results:** THPs in this study claimed that their prescribed medications had no side effects and should be researched to establish the efficacy and side effects of their herbal mixtures. A research carried out in South Africa revealed that a combination of prescribed herbal and conventional medications was effective against complications associated with antimicrobial therapy [41]. Conversely, a similar study reported that herbal medications could increase or decrease the pharmacological actions of prescribed drugs in patients with weak immune systems [42].

## Conclusion

Most of the THPs in this study (regardless of their geospatial locations urban, traditional and farmland areas) believed that diabetes is a sugar disease and referred hypertension as a disease associated with the abnormal flow of blood in a patient’s body. Some of the signs and symptoms associated with these two diseases mentioned by Tswana THPs agreed with scientific literature. Medicinal plants such as starflower *(borago officinalis),* African potato *(hypoxis hemerocallidea)* and elands pea *(senna italica)* used by Tswana THPs to manage diabetes and hypertension have been proven scientifically to be effective against them [30-32].

### What is known about this topic

- The involvement of THPs in the management of diabetes and hypertension on the African continent;
- Many people on the African continent rely on TM with the assistance of THPs because it is a cheaper alternative compared to OCM;
- Possible side effects associated with OCM encourage people to patronize TM for these chronic conditions.

### What this study adds

- Tswana THPs description of clinical features such as frequent urination, excessive sweating, frequent thirst associated with diabetes and hypertension agreed with scientific literature;
- Tswana THPs employed in this study (regardless of their geospatial locations urban, traditional and farmland areas) could be using the same treatment approaches and methods in the management of both diabetes and hypertension.
